# Trends in Radiation at a Level I Trauma Center

**DOI:** 10.7759/cureus.66471

**Published:** 2024-08-08

**Authors:** Krisha G Hidalgo, Austin Henken-Siefken, Andrew McCague

**Affiliations:** 1 College of Medicine, Western University of Health Sciences, Pomona, USA; 2 Surgery, Desert Regional Medical Center, Palm Springs, USA; 3 Trauma and Acute Care Surgery, Desert Regional Medical Center, Palm Springs, USA

**Keywords:** trauma imaging, general surgery, radiography, computerized tomography, trauma, emergency medicine, radiation

## Abstract

Introduction

Following traumatic injury, patients often require imaging modalities that subject them to large amounts of radiation. The current protocol for imaging workup in trauma patients includes plain radiographs and computed tomography (CT). Although these imaging modalities have improved quality and efficiency in trauma interventions, it is essential to understand their potential limitations and adverse effects. The large amounts of radiation from these imaging tests have raised concern for particularly vulnerable populations, including children and adolescents.

Objective

To evaluate the trends in radiation exposure in trauma assessment and imaging seen at a Level I trauma center.

Methods

This retrospective analysis used a de-identified dataset from the trauma registry at Desert Regional Medical Center Level I Trauma Center in Palm Springs, CA. Total radiation amounts were calculated based on the type of diagnostic modality. An effective radiation dose was assigned to each examination (radiograph and CT scan) based on the values provided by the American College of Radiology.

Results

There was a statistically significant positive correlation between injury severity score and effective radiation dose from imaging workup. From 2016 to 2021, radiation amount and year were found to be negatively correlated. There was a larger, statistically significant amount of radiation dosage among adult trauma patients (16.32 mSv) when compared to pediatric (6.32 mSv) and geriatric (12.67 mSv) groups.

Conclusion

Our Level I trauma center has observed a decline in radiation exposure with our current trauma protocols in place. On average, adult patients received the highest effective radiation dose to pediatric and geriatric patients. CT scans and radiography are essential tools in initial trauma workup and should be used only in appropriate patients.

## Introduction

Following traumatic injury, patients often require imaging modalities that subject them to large amounts of radiation. The current protocol for imaging workup in trauma patients includes plain radiographs and computed tomography (CT). These imaging modalities remain the gold standard for the diagnosis of blunt injuries. Emergency departments have implemented FAST (Focused Assessment with Sonography for Trauma) as an initial imaging modality to evaluate internal bleeding in trauma patients. Clinicians continue to make efforts to decrease radiation exposure to patients through increased use of non-radiographic modalities such as FAST [1-3]. Although these imaging modalities have improved quality and efficiency in trauma interventions, it is essential to understand their potential limitations and adverse effects [3]. The large amounts of radiation from these imaging tests have raised concern for particularly vulnerable populations, including children and adolescents.

According to the Centers for Disease Control and Prevention (CDC), the guiding principle of radiation safety is “ALARA,” which stands for “as low as reasonably achievable.” This principle means to avoid unnecessary radiation exposure that does not have the benefit of minimizing the harmful effects of ionizing radiation [4]. Studies have shown that radiation exposure during childhood and adolescence increases the risk of lymphoid and myeloid malignancies in adulthood [5]. According to the NIH National Cancer Institute, there is a 1 in 1,000 lifetime risk of developing cancer among people who undergo a CT scan [6]. Currently, there are limited studies that evaluate radiation exposure from diagnostic imaging tests in trauma patients. It is essential that we have a better understanding of the efficacy and outcomes of diagnostic modalities that utilize ionizing radiation to limit unnecessary exposure. The objective of this study is to evaluate the trends in radiation exposure in trauma assessment and imaging seen at a Level I trauma center.

## Materials and methods

This retrospective analysis used a de-identified dataset from the trauma registry at Desert Regional Medical Center Level I Trauma Center in Palm Springs, CA. This dataset spanned from January 3, 2016, to December 31, 2021. Our trauma center utilizes professionally trained and certified trauma registrars to populate a state database with each trauma patient. This database is then analyzed to estimate radiation exposure. The study evaluated patient demographics, imaging workup, radiation exposure, injury severity score (ISS), and discharge disposition. ISS was categorized according to the findings by Javali et al. [7]. Microsoft Excel (version 16.86, Microsoft Corporation, Redmond, WA) and GraphPad Prism (version 10.2.3, GraphPad Software, Boston, MA) were used to generate statistical analysis of the data, including regression testing and analysis of variance (ANOVA). For the tests used, it was considered statistically significant to have a value of p <0.05.

The inclusion criteria consisted of trauma patients aged 0 and above who underwent imaging workup at our Level I trauma center. Exclusion criteria encompassed individuals who had missing data, including age or ISS. Total radiation amounts were calculated based on the type of diagnostic modality. An effective radiation dose was assigned to each imaging modality based on the values provided by the American College of Radiology [8]. In this group of patients, imaging workup involved CT of the spine, head or brain, abdomen, and pelvis with or without contrast. To calculate a standard amount of contrast for CT used, we used 1 cc of contrast per 1 kg patient with an average human weight of 70 kg. Many patients underwent radiographs of the chest, spine, hip, and extremities, which were factored into the radiation dose (Table 1). 

**Table 1:** Typical effective dose values for diagnostic imaging modalities in trauma workup. *Spine includes cervical, lumbar, and thoracic regions.

Imaging Modality	Effective Dose (mSv)
Computed tomography (CT)	
Head stroke protocol	16.0
Head, with and without contrast	2.0
Brain, with and without contrast	3.2
Sinuses, with and without contrast	0.7
Maxillofacial, with and without contrast	2.0
Neck, without contrast	3.0
Spine, with and without contrast*	1.4
Chest, with and without contrast	12.4
Abdomen and pelvis, with and without contrast	15.4
Hip, without contrast	3.09
Upper extremity, with and without contrast	
Hand	0.03
Wrist	0.03
Forearm	0.05
Humerus	0.5
Elbow	0.2
Shoulder	2.06
Lower extremity, with and without contrast	
Foot	0.07
Ankle	0.07
Tibia and fibula	0.5
Knee	0.16
Femur	1.0
CT angiography	
Head, with contrast	2.0
Neck, with contrast	2.8
Chest, with contrast	6.2
Abdomen, with contrast	4.95
Lower extremity, without contrast	1.94
Radiography	
Chest	0.1
Pelvis	1
Extremity	<0.001

## Results

Our dataset from Desert Regional Medical Center Level I Trauma Center in Palm Springs, CA includes 6830 trauma patients from January 3, 2016, to December 31, 2021. Of these patients, a majority were male (58.2%) and in the adult age group between 15 and 64 years old (60.9%). Most patients identified as Hispanic or Latino (60.6%). Following the emergency department evaluation, there were 2883 admissions (42.2%) with 1371 ICU admissions (20.1%). The mean age was 54 years, with a range from 0 years to 103 years of age (Table 2). Following the emergency department, patient dispositions included admissions (42.2%), home or self-care (27.3%), ICU admissions (20.1%), surgery (6.4%), other facility (2.1%), or left against medical advice (0.9%). In this group of patients, there were 64 deaths reported (0.9%) (Table 3).

**Table 2:** Demographic data

Demographic	Number of Patients (%)
Total number of patients	6830
Gender	
Male	3976 (58.2%)
Female	2825 (41.4%)
Not reported	29 (0.4%)
Age	
Age ≥ 65 years (geriatric adult trauma)	2484 (38.9%)
Age 15-64 years (adult trauma)	4156 (60.9%)
Age ≤14 years (pediatric trauma)	190 (2.9%)
Race	
Hispanic or Latino	4137 (60.6%)
Not Hispanic or Latino	2571 (37.6%)
Other	122 (1.8%)

**Table 3:** Disposition following emergency department

Disposition from the Emergency Department	Number of Patients (%)
Admissions	2883 (42.2%)
Home or self-care	1867 (27.3%)
ICU admissions	1371 (20.1%)
Surgery (operating room)	438 (6.4%)
Other facility (including Skilled Nursing Facility, Acute Care Facility, Mental Health/Psychiatric Facility)	142 (2.1%)
Death (morgue)	64 (0.9%)
Left against medical advice	62 (0.9%)

The average ISS of total patients was 4.65 (minor ISS). The category for minor ISS (scores 1-8) included 3608 patients (57.0%). Moderate ISS category (scores 9-15) included 1911 patients (30.2%), while severe ISS category (scores 16-24) included 604 patients (9.6%). Finally, the very severe ISS category (scores of 25 and higher) only included 203 patients (3.2%). To evaluate mean differences at 5% significance, ANOVA and Tukey's post hoc test were applied for the ISS groups (Table 4).

**Table 4:** Injury Severity Score

Injury Severity Score (ISS)	Number of Patients (%)	p-Value
Average ISS of total patients	4.65 (minor ISS)	
Minor ISS (1-8)	3608 (57.0%)	
Moderate ISS (9-15)	1911 (30.2%)	
Severe ISS (16-24)	604 (9.6%)	
Very severe ISS (25+)	203 (3.2%)	
Minor vs. Moderate ISS		<0.0001
Minor vs. Severe ISS		<0.0001
Minor vs. Very severe ISS		<0.0001
Moderate vs. Severe ISS		<0.0001
Moderate vs. Very severe ISS		<0.0001
Severe vs. Very severe ISS		0.0028

**Figure 1:**
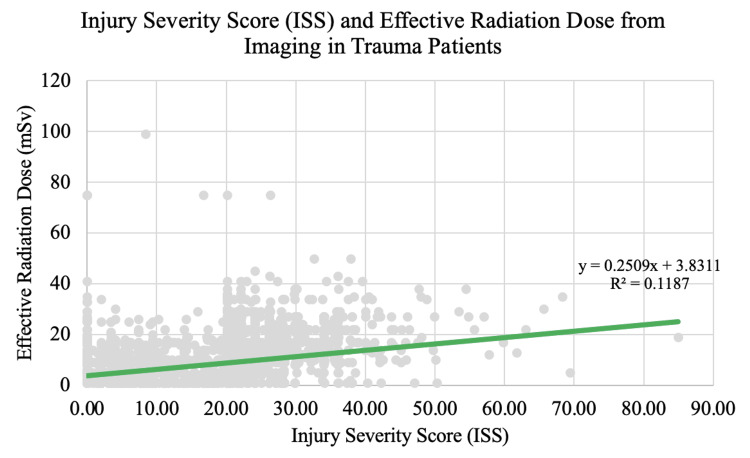
Injury Severity Score (ISS) and effective radiation dose

From the regression analysis, we found that there is a statistically significant positive correlation between ISS and effective radiation dose from imaging workup (p < 0.05). The regression equation obtained was y = 0.2509x + 3.8311 with moderate explanatory power (R^2^ = 0.1187) (Figure 1). 

**Figure 2:**
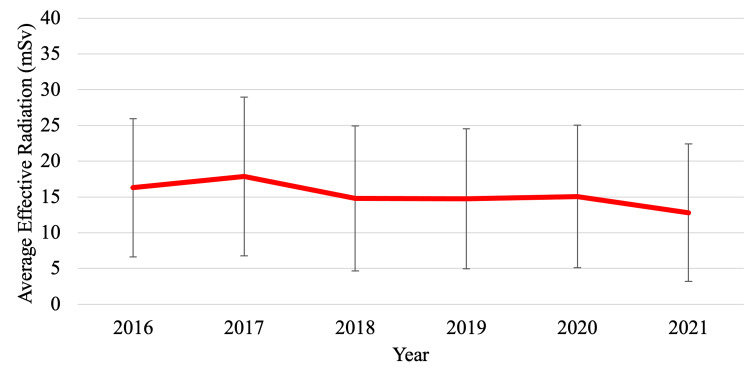
Average effective radiation from imaging workup in trauma patients (2016-2021)

From January 3, 2016, to December 31, 2021, there was an overall downward trend of average effective radiation in trauma imaging workup over time. In 2016, the average effective radiation was 16.29 mSv, followed by an increase to 17.87 mSv in 2017. From 2018 to 2019, average effective radiation decreased from 14.80 mSv to 14.77 mSv. In 2020, there was an increase to 15.06 mSv, followed by a decrease to 12.81 mSv in 2021 (Table 5, Figure 2).

**Table 5:** Year and average effective radiation from imaging workup

Year	Number of Patients	Average Effective Radiation (mSv)	Standard Deviation (mSv)	p-Value
2016	567 (8.3%)	16.285769	9.66241973	0.0004
2017	633 (9.3%)	17.8700727	11.1068551	
2018	1132 (16.6%)	14.8022906	10.1376711	
2019	1338 (20.0%)	14.7649574	9.79262966	
2020	1377 (20.2%)	15.0621002	9.95566088	
2021	1786 (26.1%)	12.8096943	9.6266204	

**Figure 3:**
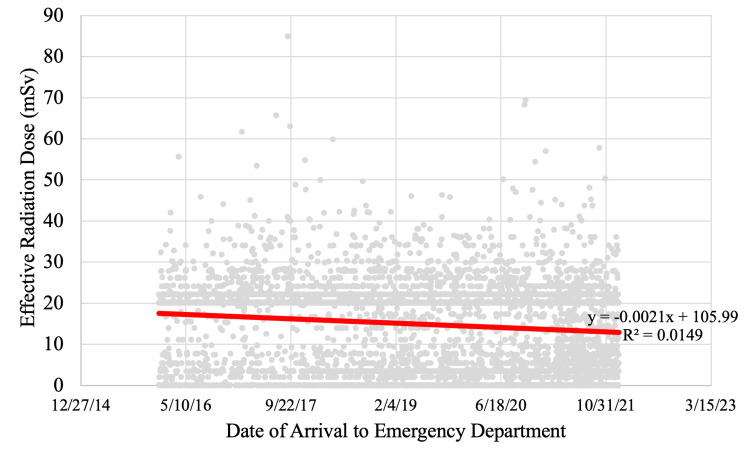
Effective radiation dose in imaging workup and date of arrival to emergency department

From the regression analysis, we found that there was a statistically significant negative correlation between the year (2016 to 2021) and imaging radiation (p < 0.0001). The regression equation obtained was y = -0.0021x + 105.99 with a relatively weak explanatory power of R² = 0.0149 (Figure 3). 

There was a larger, statistically significant amount of radiation dosage among adult trauma patients when compared to pediatric and geriatric groups. There was an average radiation amount of 16.32 mSv in the adult group, with an average amount of 6.32 mSv in the pediatric group and 12.67 mSv in the geriatric group. To evaluate mean differences at 5% significance, ANOVA and Tukey's post hoc test were applied for the age groups (Table 6).

**Table 6:** Average effective radiation dose among patient age groups

Age Group	Average Effective Radiation Dose (mSv)	Standard Deviation	Number of Patients (% of Total)	p-Value
Pediatric	6.31873158	8.30882942	2484 (38.9%)	
Adult	16.3171297	9.84419945	4156 (60.9%)	
Geriatric	12.6658474	9.73944542	190 (2.9%)	
Pediatric vs Adult Group				<0.0001
Pediatric vs Geriatric Group				<0.0001
Adult vs Geriatric Group				<0.0001

Using ANOVA statistical analysis to compare radiation doses in the age groups, with an F-statistic of 182.2 and a p-value <0.05, the results show that there is strong evidence to reject the null hypothesis and conclude that there are significant differences between the age groups (Table 7). 

**Table 7:** ANOVA comparison of radiation in pediatric, adult, and geriatric groups ANOVA, analysis of variance.

	Sum of Squares	df	Mean Square	F	Significance
Between groups	35034	2	17517	182.2	p < 0.05
Within groups	656173	6827	96.11		
Total	691207	6829			

## Discussion

The principle of ALARA guides clinicians to protect their patients from the harmful effects of ionizing radiation. Radiation is found extensively in the imaging workup of trauma patients, including radiographs and CT [4]. While imaging modalities such as CT have enhanced diagnostic accuracy in trauma assessments, there is an increasing concern for clinicians relying excessively on scan outcomes for treatment decisions, potentially resulting in inappropriate medical interventions and delayed care. Salim et al. demonstrated that ordering whole-body CT scan based on the mechanism of injury resulted in treatment changes in 20.3% of patients [9]. This trend of overuse of imaging modalities may contribute to increasing levels of radiation exposure in patients. Currently, there are limited studies that evaluate radiation exposure from diagnostic imaging tests in trauma patients.

In this group of patients, imaging workup involved CT of the spine, head or brain, abdomen, and pelvis with or without contrast. Many patients underwent additional radiographs of the chest, spine, hip, and extremities. From our analysis, we found that there is a statistically significant positive correlation between ISS and effective radiation dose (Figure 1). This finding is in line with the expectation that a patient who has undergone an injury of higher severity should undergo more extensive imaging to rule out additional internal pathologies.

From January 3, 2016, to December 31, 2021, imaging radiation and year were found to be negatively correlated (Figures 2, 3). This negative correlation between radiation amount and year may be due to several factors. One important factor may include the COVID-19 pandemic, and how lockdowns evidently limited transportation and tourism in the Palm Springs region. During the pandemic, it was found that transportation was reduced by 74%, resulting in an approximately 62% reduction in traffic collisions and fatalities [10]. We speculate that limited traffic collisions may be associated with a reduced number of trauma patients and imaging workups. Additionally, there was a new trauma group that was introduced to the Desert Regional Medical Center in 2021. During this transition, there were changes made to the trauma activation protocol that may have contributed to the change in the imaging workup of trauma patients.

Pediatric patients are an especially vulnerable population regarding radiation exposure. Young patients are found to be more sensitive to radiation and may undergo more medical diagnostic procedures throughout their lifetimes. Children are at high risk of developing cancer after radiation exposure, with an emphasis on leukemia and thyroid cancers. Our findings revealed that the lowest mean effective radiation dose was seen in pediatric patients, with an average dose of 6.32 mSv. In general, clinicians are more conservative with the use of radiation in children and adolescents, so this finding is in line with our expectations.

This paper was limited by the fact we were unable to access the specific amount of radiation undergone by each patient. Evidently, we used a method that allowed us to calculate total radiation amounts based on the type of diagnostic modality. An effective radiation dose was assigned to each examination (radiograph and CT scan) based on the values provided by the American College of Radiology. An additional limitation was that these findings of radiation trends were conducted at only one trauma center. However, there may be trends in radiation at other trauma centers that are not consistent with our findings.

Trauma patients with multiple injuries are often exposed to large amounts of radiation in initial scans. The findings from this study underscore the importance of maintaining strict adherence to ALARA in trauma assessment, especially in pediatric populations. However, it is argued that the amount of radiation from a trauma scan is negligible compared to the great risk imposed if a serious internal injury is missed. Therefore, it is imperative that we have a better understanding of the efficacy and outcomes of diagnostic modalities that utilize ionizing radiation. Radiological procedures are essential tools in initial trauma workup and should be used only in appropriate patients.

## Conclusions

In trauma patients, there was a statistically significant positive correlation between ISS and effective radiation dose from imaging workup. From 2016 to 2021, radiation dose from imaging and the year of emergency department visit were found to be negatively correlated. Additionally, there was a larger, statistically significant amount of radiation dosage among adult trauma patients (16.32 mSv) when compared to pediatric (6.32 mSv) and geriatric (12.67 mSv) groups. CT scans and radiography are essential tools in initial trauma workup and should be used only in appropriate patients.
